# YAP Overexpression in Breast Cancer Cells Promotes Angiogenesis through Activating YAP Signaling in Vascular Endothelial Cells

**DOI:** 10.1155/2022/5942379

**Published:** 2022-10-03

**Authors:** Yu Yan, Qiang Song, Li Yao, Liang Zhao, Hui Cai

**Affiliations:** ^1^Department of Breast Surgery, First Affiliated Hospital of Xi'an Jiaotong University, Xi'an, China; ^2^Department of Structural Heart Disease, First Affiliated Hospital of Xi'an Jiaotong University, Xi'an, China; ^3^Department of Thoracic Surgery, First Affiliated Hospital of Xi'an Jiaotong University, Xi'an, China; ^4^Department of Vascular Surgery, First Affiliated Hospital of Xi'an Jiaotong University, Xi'an, China

## Abstract

**Purpose:**

The YAP signaling pathway is altered and implicated as oncogenic in human mammary cancers. However, roles of YAP signaling that regulate the breast tumor angiogenesis have remained elusive. Tumor angiogenesis is coordinated by the activation of both cancer cells and vascular endothelial cells. Whether the YAP signaling pathway can regulate the intercellular interaction between cancer cells and endothelial cells is essentially unknown.

**Methods:**

The effects of YAP on tumor angiogenesis, migration, and proliferation of vascular endothelial cells were evaluated in vitro. Expression of proteins and phosphorylating proteins involved in YAP, G13-RhoA, and PI3K/Akt signaling pathways was evaluated using the Western blotting, immunofluorescence staining, and immunohistochemistry analysis. In addition, the effects of YAP on breast cancer angiogenesis were evaluated in vivo by tumor xenograft mice.

**Results:**

We showed here that conditioned media from YAP overexpressed breast cancer cells (CM-YAP+) could promote angiogenesis, accompanied by increased tube formation, migration, and proliferation of human umbilical vein endothelial cells (HUVECs). Down regulation of YAP in HUVECs reversed CM-YAP+ induced angiogenesis. CM-YAP+ time-dependently activated YAP in HUVECs by dephosphorylating YAP and increasing nuclear translocation. We also identified that both G_13_-RhoA and PI3K/Akt signaling pathway were necessary for CM-YAP+ induced activation of YAP. Besides, connective tissue growth factor (CTGF) and angiopoietin-2 (ANG-2) acted as down-stream of YAP in HUVECs to promote angiogenesis. In addition, subcutaneous tumors nude mice model demonstrated that tumors overexpressed YAP revealed more neovascularization in vivo.

**Conclusion:**

YAP-YAP interaction between breast cancer cells and endothelial cells could promote tumor angiogenesis, supporting that YAP is a potential marker and target for developing novel therapeutic strategies against breast cancer.

## 1. Introduction

Despite dramatic advances have been made in treatment of breast cancer over the last decade, the prognosis of patients with metastatic tumor is still quite poor [1]. Since tumor angiogenesis is a critical process in the metastasis and progression of cancer, it is of utmost importance to investigate the mechanism of tumor associated angiogenesis in breast cancer.

Yes-associated protein (YAP), negatively regulated by Hippo-pathway kinases, originally identified for their function in organ development have subsequently been shown to be involved in tumorigenesis [2]. The Hippo-YAP pathway is altered and implicated as oncogenic in a variety of human cancers, including human breast cancer [3]. Previous studies have focused on the direct effects of YAP in breast cancer cells, such as cancer cell proliferation, migration, and invasion. [3]. However, the effects and mechanism underlying the proangiogenic effect of YAP in breast cancer is still not fully understood [4].

Indeed, YAP activation in cancer cells has been previous showed to promote angiogenesis in cholangiocarcinoma and pancreatic ductal adenocarcinoma by directly regulating secreted cytokines [5–8] that enhanced proliferation, migration, invasion, and tube formation of endothelial cells. In breast cancer, the involvement of YAP in maintaining the phenotype and tumor-promoting functions of cancer associated fibroblasts (CAFs), ultimately encouraging tumor angiogenesis, has also been mentioned [9].

Cancer angiogenesis is coordinated by the dynamics of quiescent and activated endothelial cells (ECs) in response to multiple-growth factors and inflammatory cytokines that secreted by cancer cells, mesenchymal cells, or/and ECs [10, 11]. Activation of endothelial cells, such as proliferation, migration, invasion, and the tube formation are the hallmarks of angiogenesis. Previous studies demonstrated Hippo/YAP signaling is critical in regulating EC survival, proliferation, and migration [12, 13]. Activation of YAP caused by activation of VE–cadherin, PI3K/Akt signaling, and actin-binding protein EPS8 results in tubular network formation [10, 14].

Considering the importance of YAP activation in both cancer cells and endothelial cells could regulate angiogenesis, it is expected that YAP-YAP interaction between BCs and ECs may play an important role in tumor associated angiogenesis by regulating EC function. Here, we show for the first time that YAP overexpression in breast cancer cells can activate YAP in ECs. As a result, YAP activation leads to migration and tubular network formation of ECs, which may promote angiogenesis.

## 2. Materials and Methods

### 2.1. Reagents

The following inhibitors or reagents were used in this study: LY294002 (Enzo Life Sciences, USA), MK-2206 (Invitrogen, USA), C3 exoenzyme (Cytoskeleton, USA), and pertussis toxin (PTX; Invitrogen, USA). YAP and phospho-YAP (Ser127) were purchased from Cell Signaling (Cell Signaling technology, USA). CTGF, ANG-2, and GAPDH antibodies were from Santa Cruz Biotechnology (Santa Cruz, USA). Alexafluor secondary antibodies were from Life Technologies, USA.

### 2.2. Cell Lines and Culture

The MDA-MB-231 cells and human umbilical vein endothelial cells (HUVECs) were obtained from China Center for Type Culture Collection (CCTCC, China). All cell lines were maintained in a humidified atmosphere at 37°C with 5% CO_2_. MDA-MB-231 cells and HUVECs were cultured in DMEM/F12 (GIBCO, USA) with 15% FBS (GIBCO, USA) and 100 *μ*g/mL penicillin/streptomycin (P/S). For serum starvation, cells were incubated in growth medium without FBS or antibiotics.

### 2.3. Plasmid and RNA Transfection

To established stable cells with different YAP expression, 6-well plates were seeded with 2 × 10^5^ cell/well in 2 mL media 24 hr before transfection; cells were 80%–90% confluent. Cells were transfected with YAP CRISPR/Cas9 KO plasmid and YAP CRISPR activation plasmids using UltraCruz® Transfection Reagent (Santa Cruz, USA) according to manufacturer's instruction. After 48 hr of transfection, stable cells were selected with puromycin. For lentiviral particles transduction, Polybrene® (Santa Cruz, USA) reagent was used to introduce retroviral vectors into HUVECs according to manufacturer's instruction. All CRISPR plasmids and shRNA lentiviral particles were purchased from Santa Cruz Biotechnology (Santa Cruz, USA).

### 2.4. Conditioned Medium Preparation

MDA-MB-231 cells with different YAP expression were cultured to reach at 80%–85% confluent. The original medium was removed and cells were washed three times by PBS. Then, MDA-MB-231 cells were cultured with FBS and P/S free DMEM. After 24 hr, the supernatant was collected and labeled as YAP overexpressed medium (CM-YAP+), YAP knock down medium (CM-YAP-), and control medium (CM-Ctrl) for future studies.

### 2.5. Western Blot

Western blot analyses were conducted using standard procedures, and proteins were detected using primary antibodies and fluorescent secondary antibodies (IRDye800CW-conjugated or IRDye680-conjugated antispecies IgG) (LI-COR Biosciences, USA). The fluorescent signals were captured on an Odyssey Infrared Imaging System (LI-COR Biosciences, USA) with both 700- and 800-nm channels. Boxes were manually placed around each band of interest, and the software returned near-infrared fluorescent values of raw intensity with background subtraction (Odyssey 3.0 analytical software, LI-COR Biosciences, USA).

### 2.6. Tube Formation Assay

60-*μ*l matrigel matrix (Corning, USA) was transferred to a 96-well plate and then, incubated in 37°C incubator for 30 min. The HUVECs (2 × 10^4^ cells/well) were seeded on the matrigel matrix with different conditioned medium (CM), and incubated at 37°C for 12 hr. The tube-like structures were photographed under Qimage Retiga 2000R camera (Surrey, Canada) at 100-fold magnification, and the total tube length and total branching length from six representative fields of each group were analyzed by Image J software.

### 2.7. Plate Cloning Formation Experiment

HUVECs were digested and 300 cells of each group were seeded into 6-well plates. After 24 hr, the adherent cells were cultured with conditioned medium and the medium was changed every day. Cell culture was performed for 2-3 weeks when macroscopic apophyses were found in plates. Cells were washed and fixed with 20% methanol for 15 min. Then, fixed cells were stained with Giemsa solution for 40 min. Clones were photographed and counted using Image J software. Cloning formation rate (%) = (number of clones/number of inoculated cells) × 100%.

### 2.8. Quantitative Real-Time PCR

After transfection for 48 hr, cells were washed with cold PBS and collected in the Qiagen RLT lysis buffer (Qiagen, USA). RNA was extracted with an RNeasy mini kit (Qiagen, USA) and reverse transcribed by M-MLV reverse transcriptase. Quantitative real-time PCR was performed on a Light Cycler 480 (Roche, USA) with a SYBR Green I Master Mix (Roche, USA). mRNA abundance was normalized to GAPDH. Negative controls contained no transcript or reverse transcriptase. RNA from three separate cell pellets pretreatment was analyzed. Relative gene expression was calculated using the method given in Applied Biosystems User Bulletin No. 2. (P/N 4303859B), with nontargeting RNA-treated cells acting as the control in each dataset. Primer pairs used in this study were as follows: GAPDH: F, 5′-GAAGGTGAAGGTCGGAGT-3′/R, 5′-GAAGATGGTGATGGGATTTC-3′; CTGF: F, 5'-CTAAGACCTGTGGAATGGGC-3'/R, 5'-CTCAAAGATGTCATTGTCCCC-3'; ANG-2: F, 5'-ATCTTCCTCCAGCCCCTACAT-3'/R, 5'-GCTTCCACATCAGTCAGTTTCC-3′.

### 2.9. Immunofluorescence Staining

HUVECs were seeded in chamber slides. After treatment, cells were fixed with 4% paraformaldehyde-PBS for 15 min. Following blocking in 5% goat serum with 0.3% Triton X-100 in PBS for 60 min, cells were incubated with YAP primary antibody (1 : 100 dilution) overnight at 4°C. After three washes with PBS, cells were incubated with Alexa Fluor 488- or 555-conjugated secondary antibodies (1 : 500 dilution, Invitrogen, USA) for 2 hr at room temperature. Slides were then washed three times and mounted. Immunofluorescence was detected using a Qimage Retiga 2000R camera (Surrey, Canada) at 40× magnification.

### 2.10. Cell Migration Assays

Migration assays were conducted using Transwell plates with 8 *μ*m pore size membranes (Corning Inc., USA). After incubation for 12 hr, cells remaining in the upper side of the filter were removed with cotton swabs. The cells attached on the lower surface were fixed and stained using crystal violet and washed with water. Cells were counted with five high power fields per membrane and results were presented as the mean number of cells migrated per field per membrane. All experiments were conducted in triplicate.

### 2.11. Tumor Xenograft Experiments

All in vivo experiments were approved by the Institutional Research Committee of XXX (No. 2020-45). All mice received humane care in compliance with the Guide for the Care and Use of Laboratory Animals published by the National Institutes of Health. MDA-MB-231 cells (2.5 × 10^6^) were mixed in a 1 : 1 (v:v) ratio with Growth Factor Reduced Matrigel (BD Biosciences, USA), and the mixture was injected subcutaneously into the left (control-cancer cells) and right (YAP overexpression cancer cells) flanks of 6- to 7-week-old BALB/c nu/nu nude mice. Mice were sacrificed by cervical dislocation on day 40. Implanted tumors were extracted and the volume (measured in mm^3^) was determined using calipers and calculated using the modified ellipse formula: Volume = length × width^2^/2.

### 2.12. Immunohistochemistry (IHC) Analysis

The formalin-fixed paraffin-embedded sections (5 *μ*m thick) of the tumor tissues were analyzed by IHC using the primary YAP or CD31 antibody (1 : 100) and a biotin-conjugated secondary antibody. Four randomly selected areas were photographed at 40× magnification using a Qimage Retiga 2000R camera (Surrey, Canada). The images were analyzed using the Image-Pro Plus image analysis software (Media Cybernetics, USA).

### 2.13. Statistical Analyses

The Student's *t*-test was utilized to assess the statistical significance of the difference between two treatments. Tumor volume and CD31 positive cells between two groups were analyzed by paired *t*-test. A *P* value of less than 0.05 was considered significant.

## 3. Results

### 3.1. Overexpression of YAP in Breast Cancer Cells Promotes Angiogenesis

**Figure 1:**
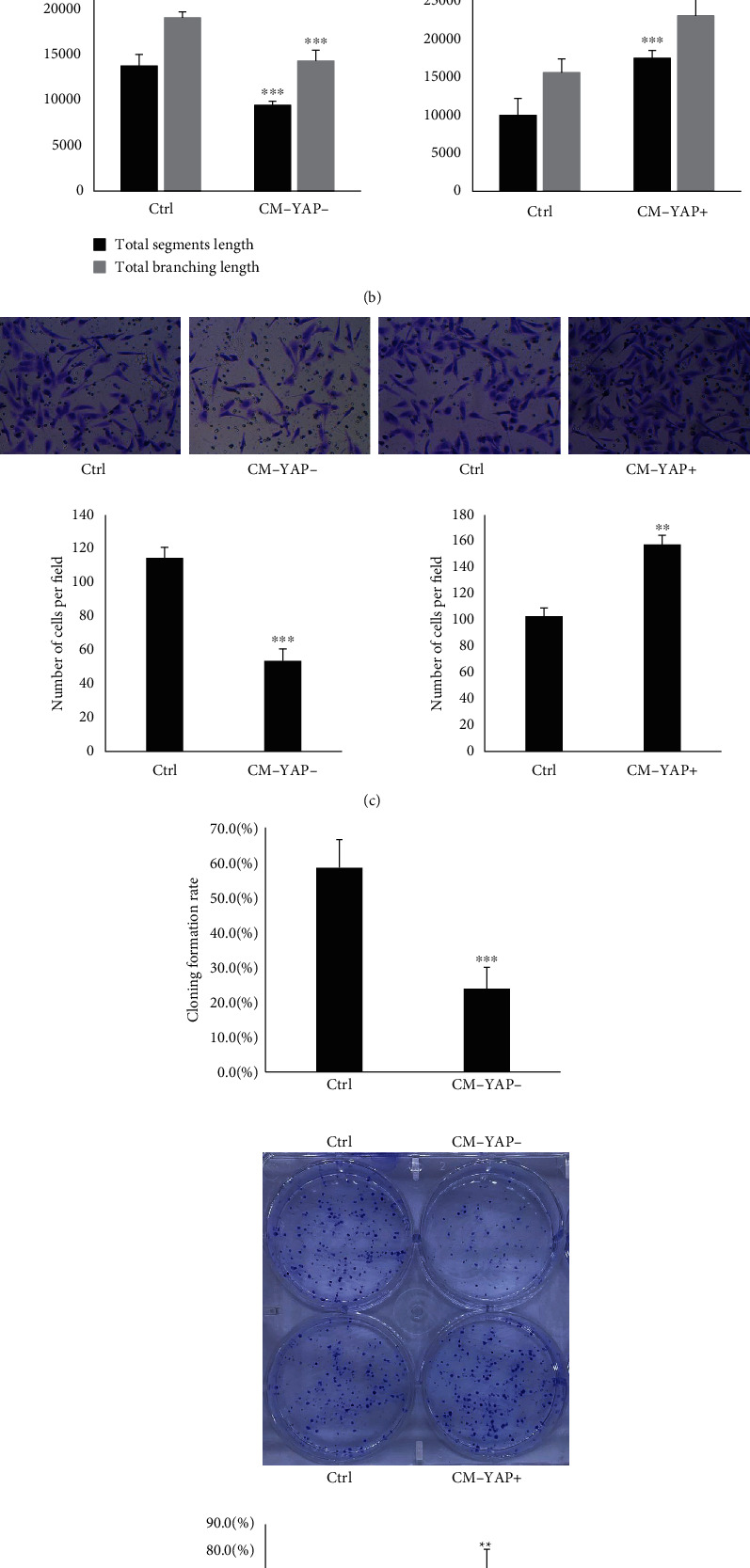
Conditioned medium from YAP overexpressed breast cancer cells promoted tube formation, migration, and proliferation of human umbilical vein endothelial cells (HUVECs). (a) MDA-MB-231 cells were transfected with YAP CRISPR/Cas9 KO plasmid (YAP KD) and YAP CRISPR activation plasmids (YAP OE), YAP expression was detected by Western blot. (b) After starved from FBS for 16 hr, human umbilical vein endothelial cells (HUVECs) were treated with conditioned medium obtained from YAP knock down (CM-YAP-) and overexpressed (CM-YAP+) breast cancer cells for 12 hr. The total segment length and total branching length from tube formation assay are quantified with means ± SD from six independent representative fields. ^∗∗∗^*p* < 0.001. (c) Migration of HUVECs with different conditioned medium treatment was conducted by Transwell assay. The results are from three independent experiments. ^∗∗^*p* < 0.01, ^∗∗∗^*p* < 0.001. (d) HUVECs were treated with different conditioned medium. Cell proliferation was tested by plate cloning formation. ^∗∗^*p* < 0.01. The cloning formation rate is quantified with mean ± SD from six independent wells. ^∗^*p* < 0.01, ∗∗∗*p* < 0.001.

While YAP was previously shown to regulate angiogenesis in other tumors [5, 6], it is not yet established whether YAP has a role in regulating angiogenesis in human breast cancer. To address whether YAP expressed in breast cancer cells (BCs) had an impact on angiogenesis, we established YAP over- and down-expression MDA-MB-231 cells (Figure 1(a)). Human umbilical vein endothelial cells (HUVECs) were cultured with conditioned media (CM) from BCs with different expression of YAP. As shown in Figure 1(b), the total tube length and total branching length were increased in CM from YAP overexpression MDA-MB-231 cells (CM-YAP+). Since migration of endothelial cells is involved in angiogenesis [15], we analyzed the effect of YAP expressed in BCs on migration of HUVECs. As results, CM-YAP+ significantly promoted HUVECs migration (Figure 1(c)). Moreover, proliferation of HUVECs was increased when cultured with CM-YAP+ (Figure 1(d)).

### 3.2. YAP Expression in HUVECs Is Involved in Mediating CM-YAP+ Induced Angiogenesis

**Figure 2:**
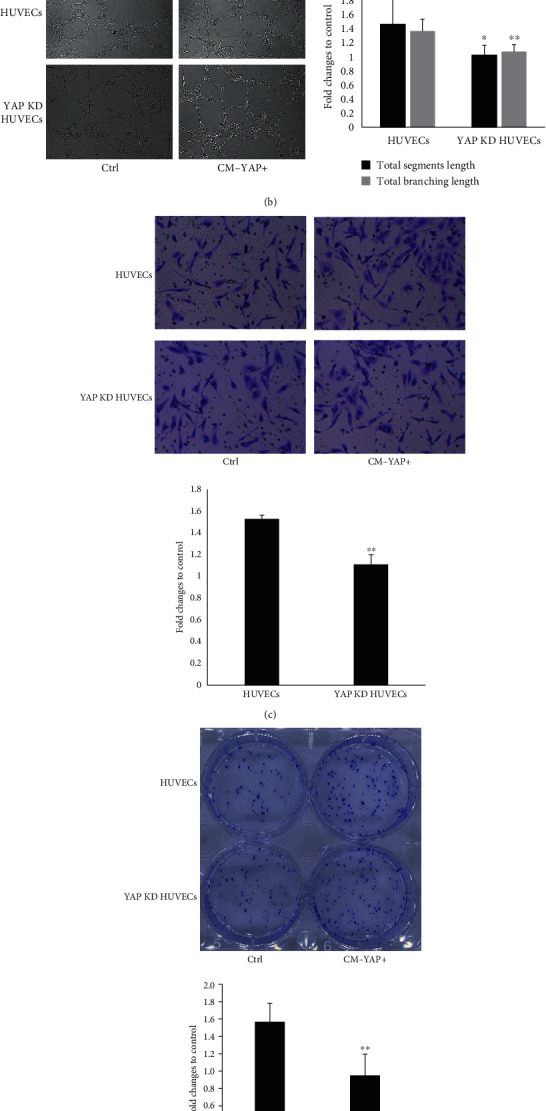
YAP expression in HUVECs involved in mediating CM-YAP+ induced tube formation, migration, and proliferation of HUVECs. (a) HUVECs were transfected with YAP CRISPR/Cas9 KO plasmid (YAP KD), YAP expression was detected by Western blot. (b) After starved from FBS for 16 hr, HUVECs, and YAP knock down (KD) HUVECs were treated with different conditioned medium obtained from breast cancer cells for 12 hr. The total segment length and total branching length are quantified with mean ± SD from six independent representative fields ^∗^*p* < 0.05, ^∗∗^ *p* < 0.01. (c) Migration of HUVECs and YAP knock down HUVECs with different conditioned medium treatment was conducted by Transwell assay. The results are from three independent experiments, ^∗∗^*p* < 0.01. (d) HUVECs and YAP KD HUVECs were treated with different conditioned medium. Cell proliferation was tested by plate cloning formation. The cloning formation rate is quantified with mean ± SD from six independent wells. ∗∗*p* < 0.01.

Hippo-YAP signaling has emerged as a key pathway that regulates endothelial cells activation which were considered as important contributions to tumor-associated angiogenesis [10, 13, 16–17]. Therefore, we tested whether YAP in HUVECs mediated CM-YAP+ induced angiogenesis. YAP expression level in HUVECs was effectively down-regulated using shRNA (Figure 2(a)). CM-YAP+ induced formation of capillary-like structures and closed loops (Figure 2(b)), as well as migration (Figure 2(c)) and proliferation (Figure 2(d)) of HUVECs were significantly reduced by YAP knock-down, supporting the functional role of YAP in HUVECs for angiogenesis induced by CM-YAP+.

### 3.3. CM-YAP+ Induces Dephosphorylation (dpYAP) and Nuclear Translocation of YAP in HUVECs

**Figure 3:**
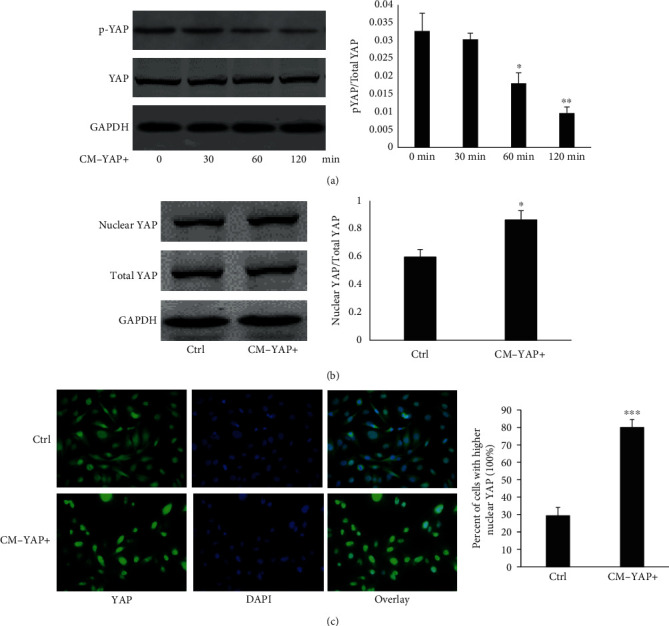
CM-YAP+ activated YAP of HUVECs. (a) HUVECs were starved for 16 hr, then, treated with CM-YAP+ for different times. Expression of total YAP and phosphorylated YAP in HUVECs were detected by Western blots. Representative results are shown from three independent experiments. ^∗^ *p* < 0.05. ^∗∗^*p* < 0.01. (b) HUVECs were starved for 16 hr, then, treated with CM for 2 hr. Expression of total YAP and nuclear YAP in HUVECs were detected by Western blots. Representative results are shown from three independent experiments. ^∗^*p* < 0.05. (c) CM-YAP+ induced YAP nuclear translocation is shown in HUVECs. Green: YAP; blue: DAPI. ^∗∗∗^*p* < 0.001.

We further tested whether conditioned media (CM) from BCs with different expression of YAP affected the dephosphorylation of YAP (dpYAP) at ser127 in HUVECs. As shown in Figure 3(a), CM-YAP+ induced dpYAP in a time-dependent manner without affecting expression of total YAP. Concomitantly, CM-YAP+ induced YAP nuclear translocation in HUVECs (Figures 3(b) and 3(c)).

### 3.4. CM-YAP+ Induced dpYAP in HUVECs Are G_13_-RhoA and PI3K/Akt Dependent

**Figure 4:**
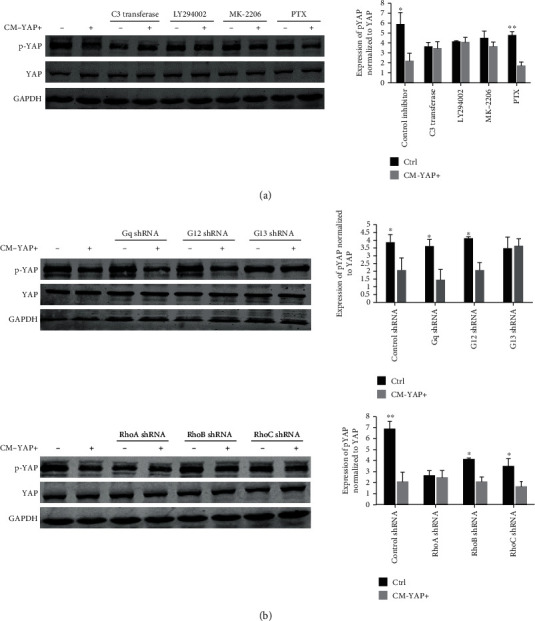
G_13_-RhoA and PI3K/Akt involved in CM-YAP+ induced dpYAP in HUVECs. (a) After starved from FBS for 16 hr, cells were pretreated with different inhibitors, LY294002 (10 *μ*M, 1 hr), MK2203 (1 *μ*M, 1 hr), and C3 (1 *μ*g/mL, 2 hr) prior to stimulation with CM-YAP+ (2 hr). pYAP and total YAP expression in HUVECs was analyzed by Western blot. ^∗^*p* < 0.05. ^∗∗^*p* < 0.01. (b) HUVECs were transfected with different shRNA for 48 hr, starved and then, treated with CM-YAP+ (2 hr). Cell lysates were analyzed by Western blot. Representative results are shown. ^∗^*p* < 0.05. ^∗∗^ *p* < 0.01.

YAP activation in endothelial cells was regulated via G protein-RhoA, and/or PI3K/Akt pathways [10, 18]. Selective pharmacological inhibitors, competitive inhibitors bind reversibly to the kinase domain of protein without affecting its expression [19–21], and reagents were used to dissect the signaling pathway leading to the CM-YAP+ induced YAP activation. CM-YAP+ induced dpYAP were completely abolished by the Rho inhibitor C3 transferase, as well as by the PI3K inhibitor LY294002 and Akt inhibitor MK-2206 in HUVECs (Figure 4(a)). Pertussis toxin (PTX, a specific inhibitor of Gi protein) and shRNA lentiviral particles of G proteins were used to determine which trimeric (large) and small G proteins were involved. CM-YAP+ induced dpYAP was insensitive to PTX (Figure 4(a)), suggesting that Gi proteins were not involved. The results from cells transfected with different shRNA lentiviral particles of G proteins showed that G_13_ and RhoA were necessary for the CM-YAP+ induced dpYAP (Figure 4(b)).

### 3.5. Connective Tissue Growth Factor and Angiopoietin-2 Acted as down-Stream of YAP in HUVECs to Promote Angiogenesis

**Figure 5:**
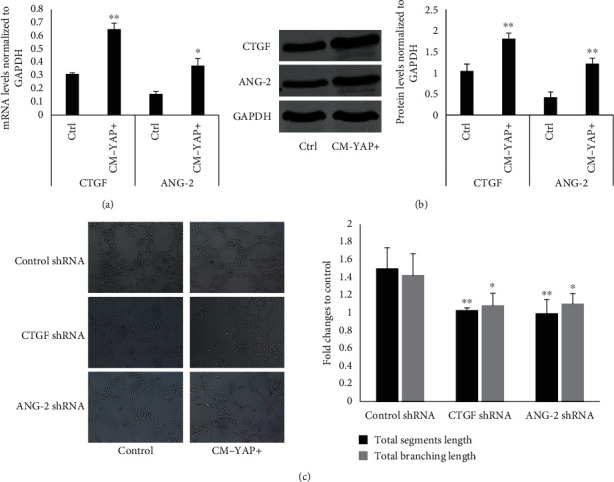
Connective tissue growth factor and angiopoietin-2 acted as down-stream of YAP in HUVECs to promote tube formation. (a) HUVECs were starved for 16 hr, then, treated with CM for 12 hr. The mRNA levels of CTGF and ANG-2 in HUVECs were determined by quantitative real-time PCR. Normalized expressions values are were quantified with mean ± SD from three independent experiments ^∗^*p* < 0.05. ^∗∗^*p* < 0.01. (b) HUVECs were starved for 16 hr, then, treated with CM for 12 hr. The protein levels of CTGF and ANG-2 in HUVECs were determined by Western blot. Representative results are shown from three independent experiments. ^∗∗^*p* < 0.01. (c) HUVECs were transfected with different siRNAs for 48 hr, starved and then, treated with CM for 12 hr. The total segment length and total branching length from tube formation assay were quantified with mean ± SD from six independent representative fields. ∗*p* < 0.05. ∗∗*p* < 0.01.

Since connective tissue growth factor (CTGF) and angiopoietin-2 (ANG-2), autocrine factors that regulates angiogenesis, have been proved as down-stream of YAP [10, 22, 23]. We tested effects of CM-YAP+ in expression of CTGF and ANG-2 in HUVECs. As shown in Figures 5(a) and 5(b), both mRNA and protein levels of CTGF and ANG-2 were significantly increased when treated with CM-YAP+ and these effects could be reversed by YAP siRNA. Furthermore, transcriptional down-expression of CTGF and ANG-2 by siRNA significantly inhibited CM-YAP+ induced angiogenesis (Figure 5(c)), demonstrating the critical roles of CTGF and ANG-2 in angiogenesis as YAP targets.

### 3.6. Overexpression of YAP in Breast Cancer Cells Promote Angiogenesis In Vivo

**Figure 6:**
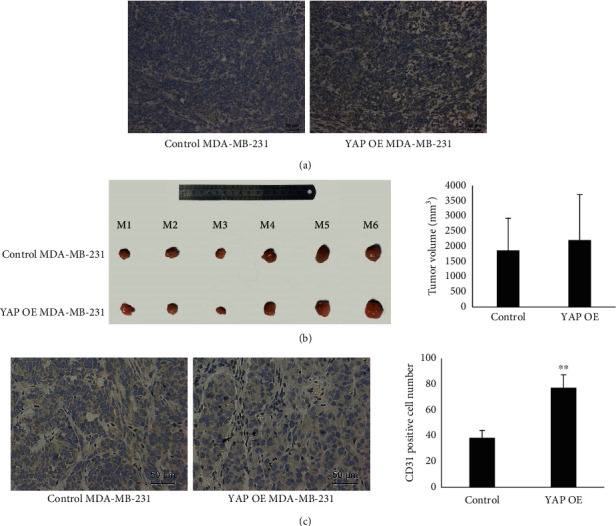
Overexpression of YAP in breast cancer cells promotes angiogenesis in vivo. (a) YAP expressed in different tumors detected by IHC. (b) Tumor volume was recorded and presented as mean ± SD. (c) CD31 positive cells (dark brown) were detected by IHC in tumors with different YAP expression. Cell number was recorded and presented as mean ± SD from six independent representative fields per tumor. ^∗∗^*p* < 0.01.

To investigate the in vivo functions of YAP in angiogenesis, we generated subcutaneous tumors in BALB/c nu/nu nude mice using MDA-MB-231 cells with different YAP expression. 40 days after tumor initiation, tumors were removed and YAP expression was confirmed by IHC (Figure 6(a)). Macroscopically, tumors with high YAP expression slightly larger than controls without statistical significance (Figure 6(b)). However, IHC showed that numbers of CD31 positive cells in YAP overexpression tumors were significantly higher than that with low YAP expression (Figure 6(c)). This result preliminarily indicated that overexpression of YAP in breast cancer cells could promote angiogenesis in vivo.

## 4. Discussion

Angiogenesis is a crucial requisite in the progression of cancers. In breast cancer, the importance of angiogenesis in the development and metastases of tumors is also well established [24]. Numerous studies have indicated many angiogenic activators and pathway that involved in the development of breast cancer. Furthermore, the use of antiangiogenic agents shows potential therapeutic effects in treatment of breast cancer. However, the clinically significant benefits of using these therapeutic agents alone have not demonstrated for the reason of the heterogeneity of breast cancer [18]. In other words, to comprehensively and systematically understand the mechanism of breast cancer-associated angiogenesis helps to discover novel biomarkers for treatment of breast cancer. Herein, we established novel angiogenic signaling pathway that overexpression of YAP in breast cancer cells activated YAP in endothelial cells resulted in migration and tube formation through G_13_/RhoA and VE-cadherin/PI3K/Akt pathway.

YAP, the transcriptional coactivators, is commonly emerged as an oncogenic factor in breast cancer through regulating multiple target genes, resulting in cancer cell proliferation and metastasis [3, 25]. However, fewer studies indicated the roles of YAP in angiogenesis of breast cancer until recently when it was shown that YAP was activated in breast cancer-associated fibroblasts and maintained the phenotype of CAFs to promote angiogenesis [9] [26, 27].In present study, we identified the direct role of YAP activation in inducing breast cancer-associated angiogenesis. In fact, many target genes of YAP have been proved to participate in cancer-associated angiogenesis through activation of ECs, especially the cytokines such as CTGF, cyr61, MFAP5, and angiopoietin-2, which could secreted by cancer cells [10, 28, 29, 5, 30]. In endothelial cells, these cytokines could stimulate multiple signaling pathways to affect the dynamics of quiescent and activated endothelial cells.

Angiogenesis is regulated by the dynamic contacts and junctions between ECs. In recent researches, VE-cadherin-dependent YAP activation was proved to regulate dynamic cell junctions and angiogenic activity of ECs in vitro and in vivo [10, 14]. Considering the proangiogenic properties of YAP activation in both cancer cells and endothelial cells, it is expected that YAP activation in BCs would activate YAP in ECs to promote cancer-associated angiogenesis. Finally, this hypothesis was verified in present study. YAP activation in ECs was regulated by mechanical stress and/or G-protein-coupled receptor signaling [13, 17, 31]. Our results revealed that both mechanical stress and G-protein-coupled receptor signaling were involved in YAP activation in ECs when treated with conditioned media from BCs that overexpression of YAP. Besides, we also identified that CTGF and angiopoietin-2 were as down-stream of YAP in ECs to promote angiogenesis, in accordance with previous studies [10, 32].

Although the significant roles of YAP-YAP activation in breast cancer-associated angiogenesis have been identified, the intercellular signal molecules between BCs and ECs that induce YAP activation in ECs have not been defined. Previous studies proved that YAP were interconnected with multiple signaling pathways initiated by soluble growth factors, such as transforming growth factor-beta (TGF-*β*), bone morphogenetic protein (BMPs), Hedgehog (Hh) and EGFR pathways [33]. Most interestingly, TGF-*β* signaling pathway could regulate YAP activation [34, 35]. Meanwhile, the TGF-*β* signaling can also been regulated by YAP activation [36, 37]. Moreover, interleukin-mediated gp130 signaling could induces the activation YAP, and increased YAP activity can upregulate gp130 signaling through a transcriptional mechanism, resulting in an amplification loop [38, 39]. Our previous study identified that YAP activation could improve extracellular expression of amphiregulin (AREG) [2], which activated EGF receptors (EGFR) signaling pathway. EGFR Signaling could also modulate YAP activation and promote cell proliferation and migration. Therefore, we tested the level changes of TGF-beta and AREG in conditional medium from breast cancer with different YAP expression. The results showed that the level of AREG was significantly higher in YAP+ CM, but the TGF-beta was not (Supplementary figure 3). We believe that more cytokines are involved in this process, which require further study.

**Figure 7:**
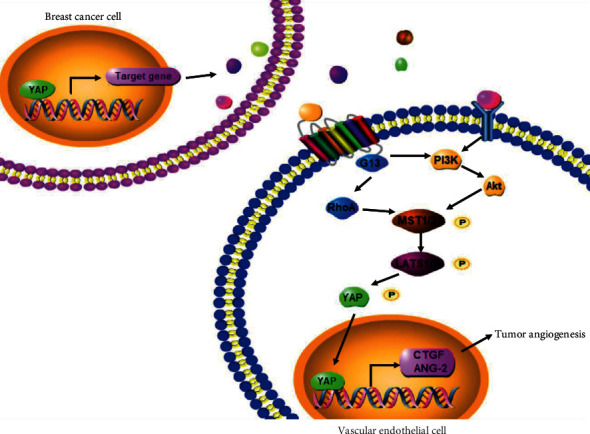
The concept map depicted the role of YAP-YAP interaction in breast cancers and vascular endothelial cells.

In conclusion, although YAP activation has been shown as a key carcinogen in driving breast cancer growth, invasion, and metastasis [40], the angiogenic role of the YAP pathways in BC is essentially unknown. The current studies not only represent the first demonstration of YAP signaling to promote angiogenesis of BC but also reveal an innovative aspect of this signaling. YAP overexpression in BC cells could activate YAP signaling in ECs, consequently promote tumor angiogenesis through both G_13_-RhoA and PI3K/Akt signaling pathway. CTGF and ANG-2 as a down-stream YAP effector to mediate tumor angiogenesis of ECs (Figure 7).

## Data Availability

The authors confirm that the data supporting the findings of this study are available within the article.

## Supplementary Materials

Supplementary Materials Supplementary figure 1: original picture of YAP blot in breast cancer with protein size markers. Supplementary figure 2: original picture of shRNAs transfection results in Figure 4. Supplementary figure 3: expression of TGF-beta (a) and AREG (b) in breast cancer cell supernatant with different YAP expression.
