# Supplementary material for: YAP Overexpression in Breast Cancer Cells Promotes Angiogenesis through Activating YAP Signaling in Vascular Endothelial Cells
Source: Anal Cell Pathol (Amst). 2022 Oct 3;2022:5942379. doi: 10.1155/2022/5942379 (PMC9550503; doi:10.1155/2022/5942379)
Supplement: Supplementary Materials — Supplementary figure 1: original picture of YAP blot in breast cancer with protein size markers. Supplementary figure 2: original picture of shRNAs transfection results in Figure 4. Supplementary figure 3: expression of TGF-beta (a) and AREG (b) in breast cancer cell supernatant with different YAP expression. [file 5942379.f1.docx]

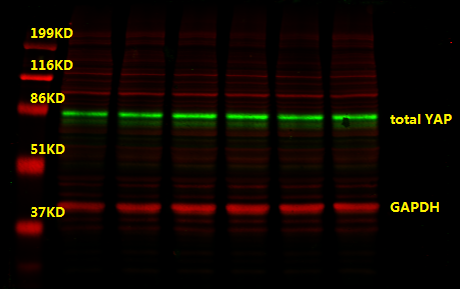


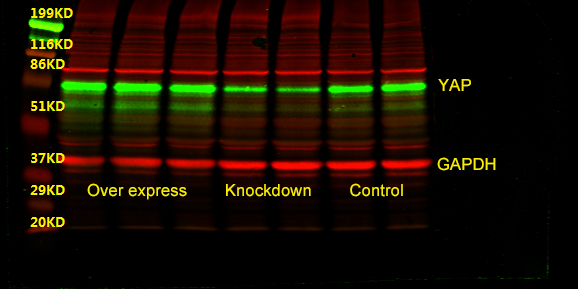


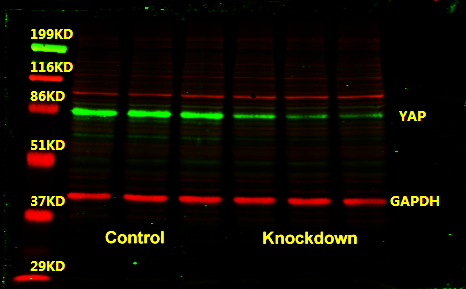


**Supplementary figure 1. Original picture of YAP blot in breast cancer with protein size markers.** Up panel was total YAP blot from normal breast cancer cells. Middle panel indicated YAP protein from breast cancer cells with different YAP expression. Down panel indicated YAP protein from HUVECs with different YAP expression.


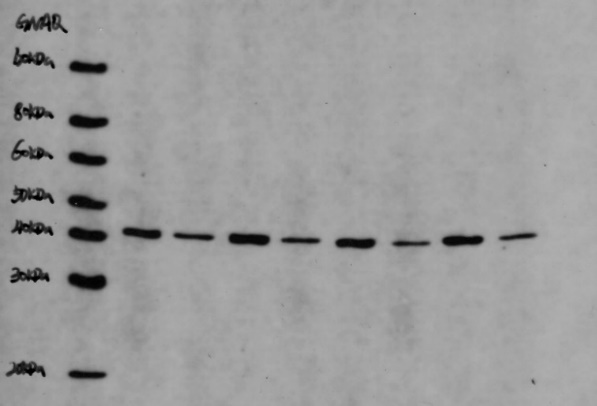

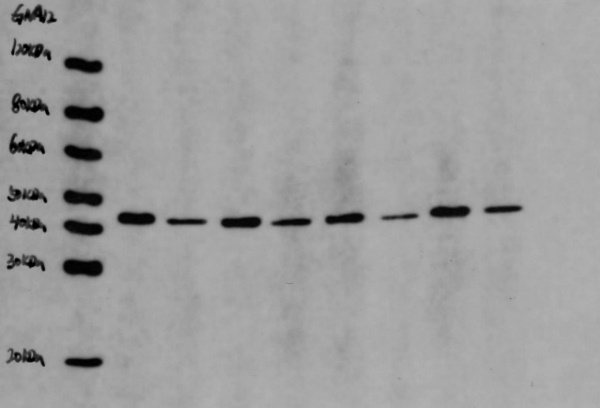


A B


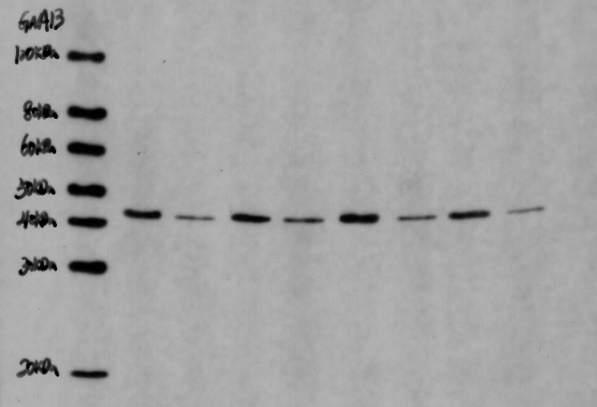

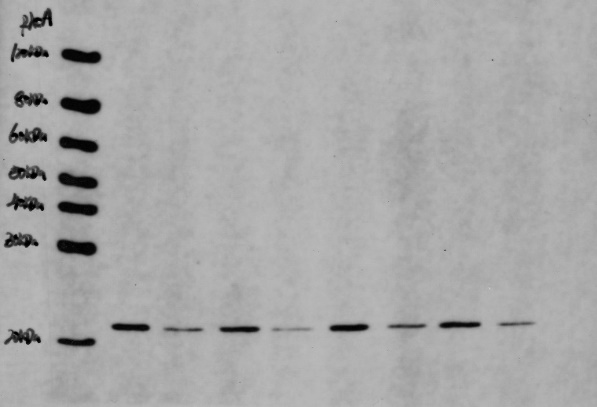


C D


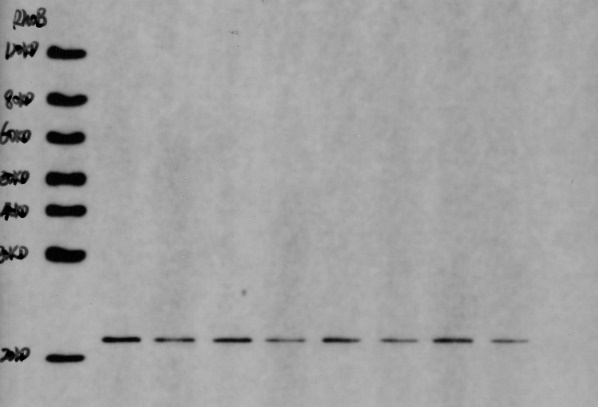

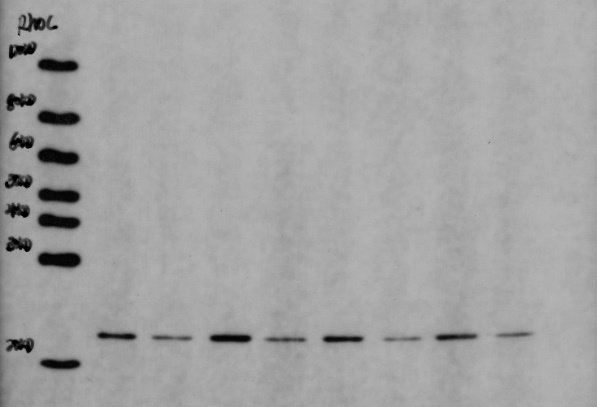


E F

**Supplementary figure 2. Original picture of shRNAs transfection results in figure 4.** HUVECs were transfected with different shRNA (A: Gq, B: G12, C: G13, D: RhoA, E: RhoB, F: RhoC), expression was detected by Western blot. Each assay was repeated three times.


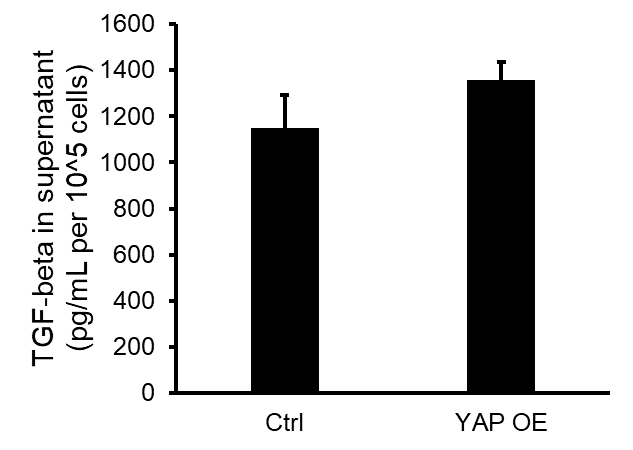

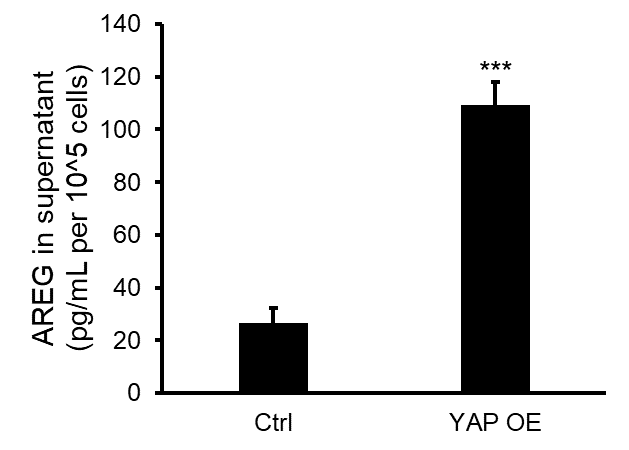


A B

**Supplementary figure 3. Expression of TGF-beta (A) and AREG (B) in breast cancer cell supernatant with different YAP expression.** Detected by ELISA. *** *p*＜0.001.
